# The convulsive syndrome in the structure of alcohol withdrawal syndrome with delirium

**DOI:** 10.1192/j.eurpsy.2021.969

**Published:** 2021-08-13

**Authors:** V. Kuzminov

**Affiliations:** Department Of Emergency Psychiatry And Narcology, SI Institute of Neurology, Psychiatry and Narcology NAMS of Ukraine, Kharkiv, Ukraine

**Keywords:** alcohol withdrawal syndrome with delirium, convulsive syndrome

## Abstract

**Introduction:**

Withdrawal states with delirium, having convulsive syndrome in their structure, are one of the most severe emergency conditions in psychiatry.

**Objectives:**

A total of 160 patients were examined with delirium alcohol withdrawal. Prognostic factors of occurrence of convulsive syndrome in the withdrawal syndrome of alcohol were studied.

**Methods:**

Clinical, psychopathological, electrophysiological.

**Results:**

It was found that the most significant prognostic factors seizures were: severe bloating condition, the duration of hard drinking, the total dose of drinking alcohol before the breakdown of consumption alcohol. Convulsive syndrome not always correlated with marked vegetative disorders in the state of withdrawal of alcohol. Convulsive syndrome that appeared after the development of delirious syndrome often indicated a more serious conditionIt is suggested that the convulsive pattern of response to the severe condition of alcohol withdrawal is formed in some young patients under the influence of endogenous factors, but is realized under certain situational conditions - long binge drinking, massive consumption o alcohol before the break of the reception of alcohol, the use of psychoactive drugs with stimulating effect. In the electrophysiological examination, there were significant differences in the group of patients with convulsive syndrome in the current admission from the group of patients with convulsive syndrome in the past and the group without convulsive syndrome in the state of abolition of alcohol.

**Conclusions:**

It is emphasized that when indicating the seizures in the state of abolition of alcohol in the past, the beginning of treatment is necessary to begin even in a state of binge drinking.

